# Advanced Sensor Technologies in Cutting Applications: A Review

**DOI:** 10.3390/s26030762

**Published:** 2026-01-23

**Authors:** Motaz Hassan, Roan Kirwin, Chandra Sekhar Rakurty, Ajay Mahajan

**Affiliations:** 1 The M.K Morse Company, Canton, OH 44707, USArakurtys@mkmorse.com (C.S.R.); 2 Department of Mechanical Engineering, The University of Akron, Akron, OH 44325, USA

**Keywords:** cutting processes, tool condition monitoring, vibration sensors, acoustic emission sensing, eddy-current sensors, machine vision, optical inspection, multi-modal sensor fusion, predictive maintenance, Industry 4.0

## Abstract

Advances in sensing technologies are increasingly transforming cutting operations by enabling data-driven condition monitoring, predictive maintenance, and process optimization. This review surveys recent developments in sensing modalities for cutting systems, including vibration sensors, acoustic emission sensors, optical and vision-based systems, eddy-current sensors, force sensors, and emerging hybrid/multi-modal sensing frameworks. Each sensing approach offers unique advantages in capturing mechanical, acoustic, geometric, or electromagnetic signatures related to tool wear, process instability, and fault development, while also showing modality-specific limitations such as noise sensitivity, environmental robustness, and integration complexity. Recent trends show a growing shift toward hybrid and multi-modal sensor fusion, where data from multiple sensors are combined using advanced data analytics and machine learning to improve diagnostic accuracy and reliability under changing cutting conditions. The review also discusses how artificial intelligence, Internet of Things connectivity, and edge computing enable scalable, real-time monitoring solutions, along with the challenges related to data needs, computational costs, and system integration. Future directions highlight the importance of robust fusion architectures, physics-informed and explainable models, digital twin integration, and cost-effective sensor deployment to accelerate adoption across various manufacturing environments. Overall, these advancements position advanced sensing and hybrid monitoring strategies as key drivers of intelligent, Industry 4.0-oriented cutting processes.

## 1. Introduction

In general, cutting is commonly performed by bandsawing, which uses bandsaw blades as the cutting tool and is often among the earliest machining operations after bulk manufacturing, producing stock sizes suitable for downstream processes [1,2]. The manufacturing of blades and saws is a precarious procedure across industries such as metalworking, woodworking, semiconductor fabrication, and composite material machining [3,4,5]. These operations demand exceptional precision and reliability, as even minor deviations in cutting performance can lead to material waste, compromised product quality, reduced tool life, and costly downtime [6,7]. In high-speed and high-load cutting environments, the complexity of interactions between the tool, workpiece, and machine introduces dynamic challenges such as vibration, thermal fluctuations, and progressive wear [8,9]. Historically, manufacturers relied on periodic inspections and operator experience to monitor tool health and process stability [10]. However, these traditional approaches are reactive, detecting problems only after they have turned into failures, a model increasingly incompatible with modern manufacturing demands for zero-defect production, predictive maintenance, and sustainable resource use [11,12].

The root of these challenges stems from the inherent variability and unpredictability of cutting processes. Blade and saw performance are affected by multiple factors such as material hardness, feed rate, cutting speed, lubrication, and machine rigidity [2,13,14]. These variables interact in complex ways, producing dynamic signals that are challenging to interpret without advanced sensing and analytics. For example, tool wear does not happen uniformly; it develops gradually, showing as subtle shifts in vibration patterns, acoustic emissions, or surface finish long before catastrophic failure [10,15]. Similarly, thermal stresses during prolonged cutting can distort blade geometry, affect dimensional accuracy, and accelerate wear [8]. Without real-time monitoring, these phenomena remain hidden until they compromise productivity and quality. To address these limitations, recent research and industrial initiatives have focused on embedding sensor technologies directly into the manufacturing process. Integrating sensors transforms conventional cutting systems into smart platforms capable of continuous condition monitoring, adaptive control, and predictive diagnostics. These sensors enable manufacturers to capture dynamic process data, identify anomalies before failure, and optimize cutting parameters, thereby improving productivity, quality, and sustainability. This shift aligns with the broader vision of Industry 4.0 and smart manufacturing, where data-driven insights underpin operational excellence.

Cutting operations are inherently harsh environments characterized by high mechanical loads, rapid thermal cycles, and abrasive contact conditions [16]. Under these circumstances, even minor deviations in tool condition can propagate into significant process instability. Sensors provide necessary visibility into these hidden dynamics, enabling manufacturers to transition from reactive maintenance to predictive and prescriptive strategies. By leveraging real-time feedback, manufacturers can dynamically adjust cutting parameters, schedule timely tool replacements, and prevent unplanned downtime, thereby contributing to cost savings, reduced downtime, and improved product quality. Recent studies have explored a variety of sensor modalities to achieve these objectives. While each sensor offers unique advantages, their collective role is to provide actionable insights into tool health and process stability. This review focuses on five key sensor types that have gained prominence in the context of cutting applications: vibration sensors [17], acoustic emission sensors [18], optical/vision-based sensors [19], force sensors [20], and eddy-current sensors [21,22]. These modalities represent complementary approaches to monitoring different aspects of the cutting process, from dynamic mechanical behavior to surface integrity and subsurface defects.

Across cutting operations, sensors are deployed to measure distinct physical quantities that reflect the underlying mechanical, thermal, and material degradation phenomena occurring at the tool–workpiece interface. Vibration sensors quantify dynamic motion in terms of acceleration, velocity, and frequency response, which are directly linked to chatter, imbalance, misalignment, and progressive wear. Acoustic emission sensors capture high-frequency elastic stress waves characterized by amplitude, energy, and event rates, enabling detection of micro-crack initiation, plastic deformation, and frictional sliding at early stages of damage evolution. Optical and vision-based sensors measure geometric and surface-related quantities such as tool edge morphology, wear land width, surface roughness, and dimensional deviations, providing direct spatial characterization of tool degradation. Eddy-current sensors infer changes in electromagnetic impedance caused by induced eddy currents, which are sensitive to material conductivity, lift-off distance, and subsurface defects such as fatigue cracks and thermal damage. Force sensors directly measure cutting mechanics through force and torque components and their temporal variations, providing physically interpretable indicators of chip formation, frictional conditions, tool wear progression, and process stability, and thereby enabling reliable detection of tool degradation, breakage, and chatter onset. It is important to note that no single sensor modality can fully capture the multi-physics nature of cutting processes. Many sensors can respond to multiple physical quantities simultaneously, while accurate diagnosis of complex phenomena such as chatter, wear progression, and fracture often requires combining complementary measurements across different sensing principles. Consequently, recent research has increasingly focused on multi-sensor and hybrid monitoring strategies that integrate mechanical, acoustic, optical, and electromagnetic information to improve diagnostic accuracy and robustness under varying cutting conditions. By mapping these measured physical quantities to specific cutting phenomena, sensor technologies enable complementary and multi-scale monitoring of tool condition and process stability.

Section 2 examines vibration sensors. Section 3 discusses acoustic emission sensors. Section 4 explores optical/vision-based sensors. Section 5 covers eddy-current sensors. Section 6 details force sensors. Section 7 expands on multi-sensor fusion-based approaches. By organizing the discussion around these five sensor types, this review aims to provide a comprehensive understanding of how advanced sensing technologies are being integrated into cutting processes. The goal is not only to highlight the technical principles behind each modality, but the main aim is to examine their practical implications for improving process reliability, reducing waste, and enabling predictive maintenance strategies.

Although a significant portion of the referenced literature originates from turning and milling operations, the underlying physical mechanisms governing material removal, tool–workpiece interaction, and wear progression share strong similarities with sawing processes. In all cases, cutting involves cyclic mechanical loading, frictional contact, heat generation, and progressive tool degradation, which manifest as measurable changes in vibration, acoustic emission, cutting forces, and surface geometry. These commonalities enable sensing and monitoring strategies developed for turning and milling to be meaningfully adapted to saw blades and circular saws. However, notable differences must be considered. Sawing typically involves multi-tooth engagement with distributed cutting forces, intermittent contact, thinner cutting edges, and flexible blades, leading to unique dynamic behaviors such as blade flutter, tooth-level load variation, and higher sensitivity to tension and alignment. Unlike rigid turning and milling tools, saw blades exhibit pronounced elastic deformation and tension-dependent stability. Consequently, while signal interpretation frameworks remain transferable, sensor placement, frequency content, and diagnostic thresholds must be adapted specifically for sawing applications.

## 2. Vibration Sensors

**Figure 1:**
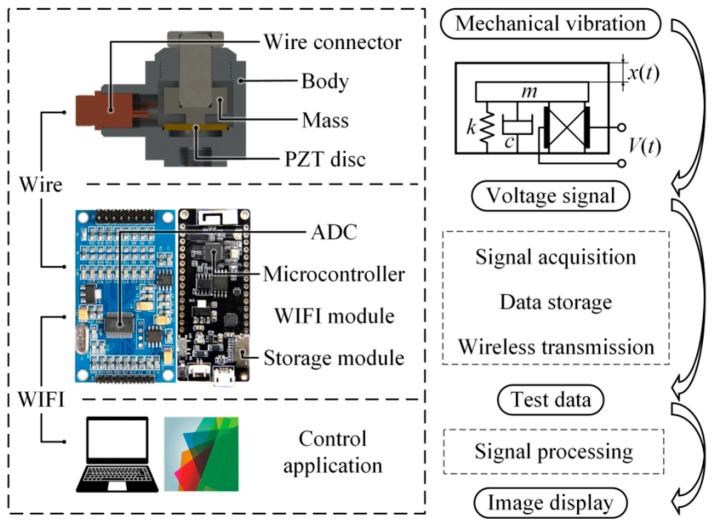
System components and signal flow for the IoT-based vibration sensing platform. The diagram depicts the mechanical design of the vibration transducer, the electronic hardware for signal acquisition and wireless transmission, and the sequential stages of data transformation from raw mechanical vibration to end-user image display [32].

Vibration sensors are critical components in modern manufacturing systems, particularly in processes involving high-speed cutting tools such as bandsaws. These sensors operate by detecting oscillatory motion and converting it into electrical signals, enabling precise monitoring of machine health and operational stability [23]. The composition of these sensors typically involves a sensing element, such as a piezoelectric crystal or a micro-machined silicon cantilever, housed within a protective casing to withstand industrial environments (Figure 1). Common technologies include piezoelectric sensors, which generate voltage under mechanical stress and are ideal for high-frequency detection; capacitive sensors, which measure changes in capacitance due to displacement for low-frequency applications; and microelectromechanical system (MEMS)-based sensors, which leverage micro-electromechanical systems for compact and highly sensitive designs. In cutting operations, vibration sensing provides measurements of acceleration, velocity, or displacement that reflect dynamic interactions between the blade, spindle, and workpiece. These physical quantities capture chatter, periodic tooth engagement forces, misalignment, and progressively increased broadband energy associated with tool wear and frictional changes. By capturing dynamic responses, vibration sensors provide essential insights into mechanical conditions such as imbalance [24,25], misalignment [26,27], and wear [28,29], making them indispensable for predictive maintenance and quality assurance in industrial environments. In the specific process of developing a bandsaw or blade, this vibration sensor technology is helpful as it allows engineers to map the harmonic response of the blade material and tooth geometry under varying load conditions. By identifying the specific frequencies at which resonance occurs, developers can refine the blade’s physical form to dampen unwanted oscillations, thereby increasing the fatigue life of the steel and ensuring precision of the cut. Currently, variable pitch and long tooth patterns are used to reduce the harmonics and vibrational effects during the cutting process [30,31].

**Figure 2:**
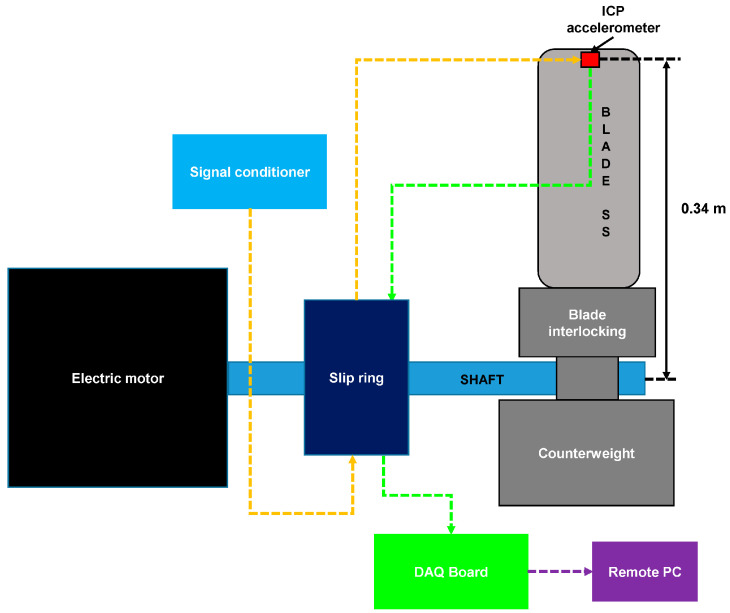
Schematic of an experimental testbed for rotating blade vibration monitoring. The setup includes a 0.34 m blade mounted on a motor-driven shaft with a counterweight for balance. A tip-mounted ICP accelerometer (vibration sensor) captures dynamic responses, which are transmitted via a slip ring assembly to a signal conditioner and DAQ board for remote data processing and analysis. Different colored arrows represent connection points of data [36].

Vibration sensing, particularly via compact MEMS accelerometers and piezoelectric sensing elements, enables tool condition monitoring and predictive maintenance (Figure 2). Vibration is measured near the cutting system, providing information linked to the tool state without intervening in the cutting zone. A recent study validates how indirect monitoring, relying on sensors situated around the cutting area, infers wear progression and decision making in machine operations [12]. Domain studies in turning and milling operations further operationalize this linkage by using vibration amplitude/acceleration patterns as inputs to data-driven wear prediction models, such as artificial neural networks (ANN)-based wear prediction in turning [33]. Similarly, there are literary frameworks that treat vibration as a primary sensor modality alongside force and other signals in wear prediction [34]. In parallel, a low-cost IoT predictive maintenance framework was recently introduced, utilizing vibration monitoring via MEMS accelerometers and explicitly highlights how resonant-operated MEMS transducers can improve sensitivity for low-frequency condition monitoring, aligning sensor selection with the frequency bands where many machine-health degradations manifest [35]. At the integration level, research has directly characterized MEMS accelerometer accuracy limitations in rotating reference frames, an issue that is practically pertinent for industrial spindles and rotating fixtures, by analyzing amplitude/phase accuracy and clock-related effects that can bias vibration estimates used downstream for diagnosis [36]. Process-monitoring case studies evaluate “on-rotor” multi-axis sensing performance for machining monitoring and motivate the use of piezoceramic (piezoelectric) components due to their stiffness/sensitivity and miniaturization/integration potential for vibration control and monitoring in cutting operations [37]. Complementing these devices and integration facing contributions, manufacturing-focused studies increasingly emphasize that the value of vibration sensors is realized through robust inference under non-stationary cutting conditions. For example, a MEMS-based milling monitor for turbine blade manufacturing explicitly notes that vibration is a strong tool-condition indicator but can be confounded by machine stiffness, alignment, tool–work interactions, and changing cutting conditions, making attribution of vibration sources central to reliable monitoring [38]. Accordingly, recent work has shifted from rule-based feature extraction toward machine learning approaches for tool condition monitoring using vibration signals (e.g., CNC gear cutter monitoring) to handle nonlinear relationships between vibration patterns and wear [39]. This shift directs a general data-driven drilling tool fault diagnosis pipeline that integrates vibration-based monitoring with machine learning for real-time, scalable predictive maintenance [40]. Saw-related manufacturing applications demonstrate that vibration-sensor-driven analytics are being extended beyond conventional machining. Machine-learning modeling of circular sawing machine vibration during cutting provides an example of how vibration data can be used to assess and predict system vibration behavior under process/material variability, reinforcing the role of vibration sensing as an actionable input to predictive decision-making in cutting-based manufacturing [41].

Collectively, these recent studies present a clear trend of convergence of vibration sensing, IoT connectivity, and AI-driven analytics to redefine bandsaw manufacturing. The emphasis is shifting from reactive maintenance to proactive, data-driven strategies to enhance productivity and reduce operational risks. Despite these advancements, several limitations persist with vibration sensors. One major challenge is signal complexity, as vibration data often contains noise from multiple sources, making accurate fault diagnosis difficult [42]. Sensor placement is critical as improper positioning leads to inaccurate readings and ineffective monitoring [43,44]. Another limitation is the cost of implementation, particularly for small and medium-sized enterprises, where deploying IoT-enabled sensor networks could require significant investment. Environmental factors such as temperature fluctuations and dust can also affect sensor accuracy and longevity. While machine learning models have improved predictive capabilities, they require large datasets for training, which may not always be available in niche manufacturing setups. Future research aims to bridge this through the development of robust sensor fusion techniques combining vibration data with other modalities such as acoustic emission, thermal imaging, and force measurements to improve diagnostic accuracy. Miniaturization of sensors using MEMS technologies will enable easier integration into a compact cutting system. AI-driven adaptive control algorithms should explore real-time optimization of cutting parameters based on sensor feedback. Another promising direction is the use of digital twins, where virtual models of bandsaw systems are continuously updated using sensor data to simulate and predict cutting system behavior, including blade vibration response, wear progression, and stability limits, under varying operating conditions such as cutting speed, feed rate, material hardness, blade tension, and lubrication state. Efforts should be made to reduce the cost and complexity of IoT-enabled sensor networks, making them accessible to small-scale manufacturers and accelerating the adoption of smart manufacturing practices globally. While vibration-based monitoring is widely adopted due to its low cost and ease of integration, reported results vary significantly across studies due to differences in sensor placement, machine rigidity, and cutting configurations. Many vibration-based models are trained under narrowly defined operating conditions, limiting their generalizability to industrial environments with variable materials and process parameters. Moreover, vibration signals often reflect multiple concurrent phenomena, making it difficult to isolate wear-related features without complementary sensing modalities.

## 3. Acoustic Emission (AE) Sensors

**Figure 3:**
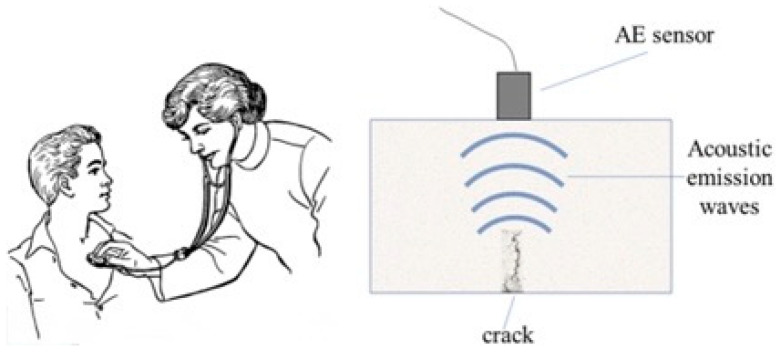
Similar methodologies of use between the stethoscope and the AE sensor [46].

Acoustic emission (AE) sensing is a highly sensitive non-destructive evaluation and process monitoring technique that detects transient elastic stress waves generated by localized, rapid energy releases within a material subjected to mechanical loading, including phenomena such as micro-crack initiation (Figure 3) and propagation, plastic deformation at the cutting edge, frictional sliding at the tool–workpiece interface, and adhesive or abrasive wear mechanisms [45,46]. Unlike conventional vibration sensors, which primarily measure global structural responses and oscillatory motion in the low- to mid-frequency range, AE sensors are designed to capture ultrasonic stress waves typically spanning 20 kHz to 1 MHz, enabling the detection of highly localized, short-duration events that are directly linked to microstructural damage and incipient failure mechanisms. Resulting descriptors of AE sensing including amplitude, energy, and event counts provide high-frequency sensitivity to identify subtle changes in material behavior and cutting conditions that precede measurable increases in vibration, force, or temperature. In manufacturing processes involving cutting blades such as bandsaws, circular saws, and continuous sawing systems, AE sensing has been extensively employed for early-stage tool wear detection, chatter onset identification, and real-time process monitoring, as it provides direct insight into tooth-level degradation, edge chipping, and frictional instability. By capturing microscopic damage mechanisms before they evolve into macroscopic wear, surface degradation, or catastrophic blade failure, AE sensing enables more accurate predictive maintenance, improved cutting stability, and data-driven optimization of cutting parameters, making it a critical sensing modality in modern high-performance and smart manufacturing environments.

**Figure 4:**
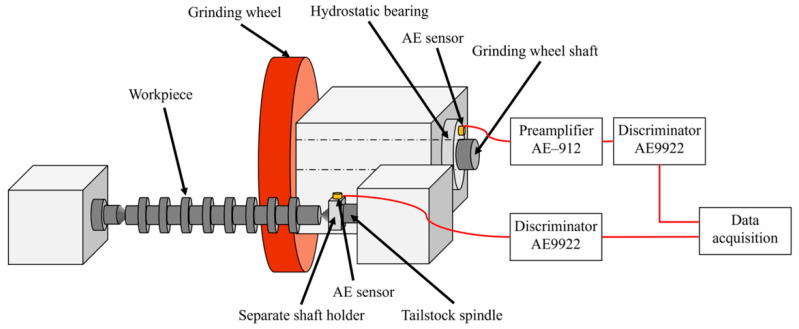
Example of an AE sensor used for fault detection in a grinding process [60]. The cylindrical grinding machine: GE4Pi CNC cylindrical grinding machine (JTEKT Corporation, Aici, Japan). Workpieces were made of JIS SCr420 similar to AISI 5129 (carborized and then quenched). FGS700-soluble cutting fluid (Yushiro Chemical Industry Corporation, Tokyo, Japan), Both AE sensors are S9225 AE sensors (Physical Acoustics Corporation, West Windsor Township, NJ, USA with frequency bandwith 0.3–1.8 MHz). AE9922 discriminator (NF Corporation, Kanagawa, Japan). AE-912 preamplifier (NF corporation, Kanagawa, Japan). Aquired AE signals were recorded at 5 MS/s using DL850 ScopeCorder (Yokogawa Electric Corporation, Tokyo, Japan).

Recent studies demonstrate that AE sensing has become a technically robust and industrially viable solution for monitoring continuous cutting processes, where sustained tool–workpiece interaction, cyclic loading, and progressive blade wear dominate system dynamics (Figure 4). At the fundamental signal level, Maia et al. showed that AE sensors operating in the ultrasonic frequency range are highly sensitive to localized energy releases associated with frictional sliding, plastic deformation, and micro-crack initiation at the cutting edge, enabling earlier wear detection than conventional vibration-based methods [47]. Complementing this, Zhang et al. demonstrated that AE time–frequency representations, when processed using deep learning models, can reliably distinguish multiple tool wear states under varying cutting conditions, highlighting AE’s suitability for intelligent monitoring of non-stationary cutting processes [48]. In cutting environments closely analogous to sawing, such as wood machining, Dado et al. reported strong correlations between AE parameters, including RMS amplitude, hit count, signal energy, blade sharpness, feed rate, and cutting stability, confirming AE’s robustness for long-duration, continuous cutting operations [49]. Advances in signal processing have further strengthened AE-based diagnostics. Studies demonstrate how wavelet-based and short-time Fourier transform (STFT) analyses can isolate wear and chatter-related frequency bands, allowing differentiation between abrasive wear, edge chipping, and unstable cutting regimes [50,51]. The integration of AE sensing with data-driven models has accelerated in recent years, with Diaz et al. and Penicaud et al. showing that machine learning and deep learning architectures trained on AE spectral features outperform traditional threshold-based methods in classifying cutting states and predicting tool degradation [52,53]. From a hardware and deployment perspective, Uematsu et al. developed wireless AE sensor systems with integrated signal conditioning, addressing practical challenges related to sensor placement, cabling, and durability in harsh industrial environments such as sawing machines [54]. The applicability of AE sensing to predictive maintenance was further reinforced by Hidle et al., who demonstrated AE’s ability to detect subsurface micro-cracks and incipient damage well before visible wear occurs, a capability critical for preventing sudden blade failure in bandsaw systems [55]. System-level integration was explored by Matheus et al. and Nasir and Banaras, who embedded AE sensing into an IoT-enabled monitoring framework for real-time tool health assessment and cloud-based analytics, aligning AE technology with Industry 4.0 manufacturing paradigms [56,57]. Additionally, Zaman et al. and Nasir et al. showed that fusing AE with vibration signals improves diagnostic reliability by capturing both high-frequency fracture events and low-frequency structural responses, which is particularly beneficial for identifying chatter and instability in continuous cutting [58,59]. Collectively, these studies firmly establish acoustic emission sensing as a cornerstone technology for advanced bandsaw monitoring and predictive maintenance in modern smart manufacturing systems.

The current AE sensor in saw manufacturing faces several technical constraints. Although AE sensing consistently demonstrates superior sensitivity to early-stage damage, most studies rely on laboratory-scale setups with controlled noise conditions and optimized sensor placement. High data rates and signal interpretation complexity remain barriers to real-time industrial deployment, and comparative studies evaluating AE performance against lower-cost alternatives under identical conditions are still limited. Overlapping fault signatures and signal complexity often make diagnosis challenging, as vibration and acoustic emission data require advanced filtering and feature extraction. Real-time processing bottlenecks, particularly for high-frequency AE signals, limit adaptive control when relying on cloud-based systems. Sensor durability under harsh conditions and the absence of standardized communication protocols hinder scalability and increase maintenance costs. Predictive models also lack cross-platform generalizability, requiring extensive retraining for different machine configurations. Future work aims to develop universal standards for sensor interoperability, enabling seamless integration across platforms. Research into hybrid AI models combining physics-based and data-driven approaches can improve fault classification accuracy while reducing data dependency. Incorporating edge computing and federated learning will address any latency and privacy concerns, while energy-harvesting sensor designs could improve sustainability. Leveraging digital twins and augmented reality interfaces will allow real-time visualization and simulation of corrective actions, accelerating the transition toward autonomous, self-optimizing manufacturing systems.

## 4. Optical/Vision-Based Sensors

**Figure 5:**
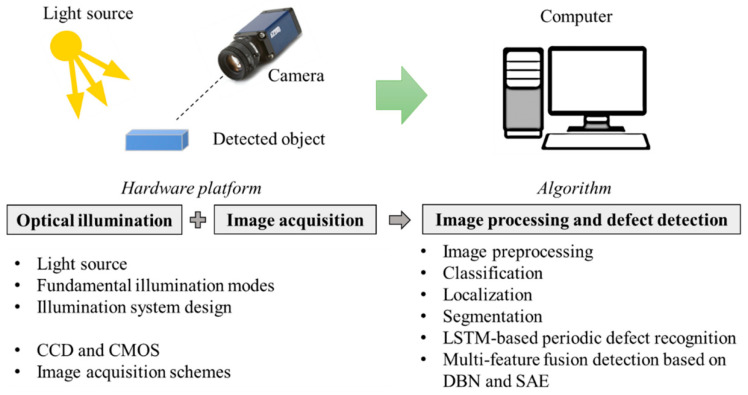
An illustration of a visual inspection system architecture and the process for defect detection [64].

Optical and vision-based sensing technologies have become increasingly integral to modern manufacturing environments, particularly in cutting processes, where geometric accuracy and surface integrity are critical to performance and product quality. These systems employ a range of sensing modalities, including high-speed industrial cameras, laser triangulation, structured-light projection, laser line scanning, and hyperspectral or multispectral imaging, to acquire high-resolution two-dimensional images and three-dimensional surface profiles of cutting tools and workpieces [61,62,63]. Unlike vibration or acoustic emission sensors, which infer tool condition indirectly through dynamic signal responses, optical and vision-based sensors provide direct, spatially resolved measurements of tool geometry, edge condition, surface roughness, and wear progression (Figure 5). Vision-based measurements capture geometric and surface-related quantities including flank wear land width, crater wear, chip morphology, and tooth alignment. These observable features directly characterize material removal and wear progression, offering spatial resolution beyond what can be inferred from purely dynamic sensors. Their non-contact nature allows continuous, real-time inspection without interfering with the cutting process, making them well suited for high-speed sawing operations where physical sensor placement is challenging. In cutting applications, vision-based systems are particularly effective at detecting blade misalignment, tooth chipping, pitch irregularities, surface defects, and dimensional deviations, enabling rapid identification of process instabilities and tool degradation. When integrated with machine vision algorithms and AI-based image analysis, these systems support automated quality control and closed-loop process monitoring, significantly reducing reliance on manual inspection while improving inspection consistency, repeatability, and overall production efficiency in advanced manufacturing lines.

**Figure 6:**
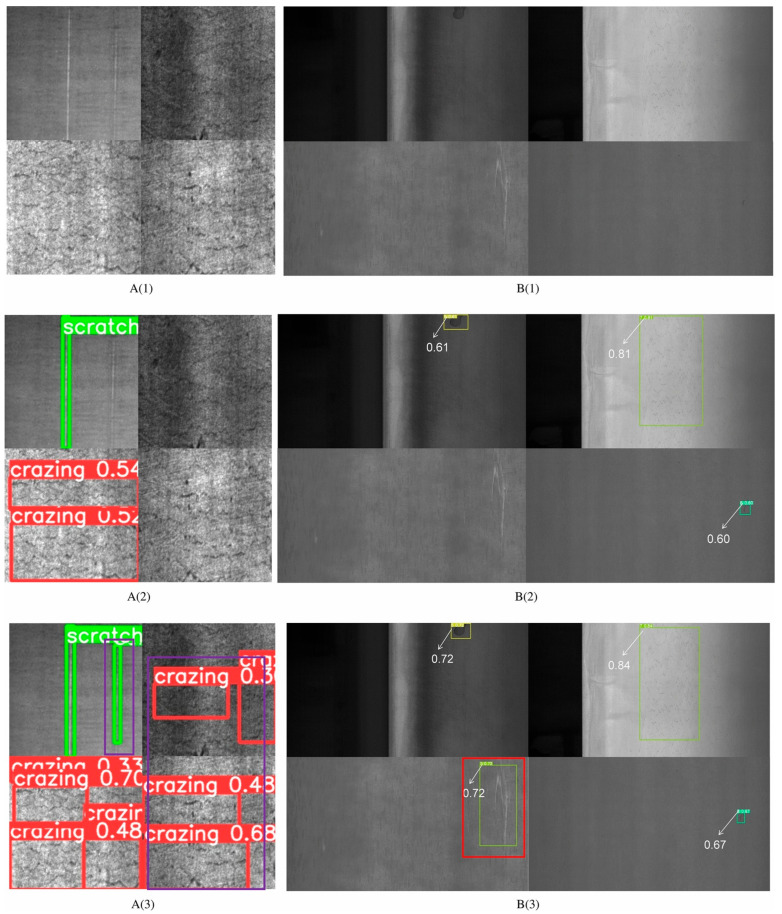
Images on the left column A(1–3) represent Northeastern University Surface Defect Database for Detection (NEU-DET) images while images on the right column B(1–3) represent GongChang Surface Defect Database for Detection (GC10-DET) dataset. Comparison of defect detection performance across deep learning models (1st row: original image, 2nd row: YOLOv9c, and 3rd row: EPSC-YOLO), illustrating localization of surface defects and wear features on the cutting tool edge and flank region, where chipping, abrasion, and surface damage typically initiate [72].

Recent studies have increasingly leveraged optical and vision-based systems for direct, non-contact monitoring of cutting tools and workpiece conditions during manufacturing processes, offering geometric and surface information that complements traditional indirect sensor modalities (Figure 6). A notable development in this area is the integration of microscope-based on-machine vision systems with CNC milling and cutting machines to capture high-resolution images of tool edges during operation [65]. These systems, when combined with deep learning classification models such as Efficient-Net, have demonstrated the ability to identify multiple tool wear states (e.g., flank wear, adhesion, chipping) with high classification accuracy, thereby enabling real-time tool health assessment without interrupting machining operations [66]. Building on this, recent work has explored robust tool wear monitoring methodologies in micro-milling by applying advanced image segmentation and reconstruction techniques to mitigate challenges such as uneven illumination, low pixel contrast, and complex tool geometries, resulting in more accurate extraction of wear features for classification with convolutional neural networks [67]. Complementary efforts have systematically compared traditional and deep learning-based computer vision methods for quantifying wear on gear cutting tools, affirming that modern vision algorithms can detect and measure wear patterns with sufficient detail for predictive maintenance and quality control [68]. State-of-the-art image processing for tool condition monitoring shows that both direct image-based approaches (synchronizing cameras with cutting tools to measure wear zones) and indirect image processing techniques (analyzing chip geometry and surface features) extend the capability of vision systems to assess tool and workpiece conditions across a variety of machining operations [69]. Scalable vision systems have also been developed using consumer-grade and low-cost camera hardware coupled with real-time image processing, demonstrating that even accessible imaging solutions can detect tool breakage and near-edge fractures during cutting processes, making vision-based monitoring more practical and cost-effective for industry deployment [70]. In addition to tool wear quantification, emerging studies present improved lighting-robust vision techniques and explainable artificial intelligence approaches that maintain high performance under variable industrial lighting environments, a common key challenge for industrial vision integration [71]. Vision-based methods are being extended to workpiece surface and dimensional inspection, where deep learning models classify surface textures and dimensional deviations resulting from tool wear and cutting instability, enabling automated quality assurance in machining workflows.

Advancements in inspection, image segmentation, deep learning, and real-time vision integration highlight the maturing role of vision-based sensing in the cutting process, where direct imaging provides actionable, high-resolution insights into tool condition, surface roughness, and process health that are essential for predictive maintenance, adaptive control, and automated quality control in advanced manufacturing systems. Despite their growing adoption and integration, vision-based and optical sensing technologies face limitations in cutting environments. One major challenge is environmental sensitivity, as factors such as dust, coolant splashes, and variable lighting conditions can degrade image quality and compromise detection accuracy. To mitigate this, industrial vision systems have successfully employed air-knife nozzles or transparent protective windows with continuous air purging to prevent dust and coolant accumulation on lenses, combined with adaptive LED ring illumination and image normalization algorithms to maintain reliable defect detection in contaminated cutting environments. High-speed cutting operations introduce motion blur and vibration-induced distortions, which require advanced image stabilization and high-frame-rate cameras, increasing system complexity and cost. Another limitation is computational demand, as real-time image processing for defect detection and adaptive control often requires powerful hardware or edge computing solutions, which may not be feasible for small-scale manufacturers. Integration challenges persist when combining vision systems with existing CNC or IoT architectures, due to a lack of standardized communication protocols. Current AI-driven vision models also suffer from data dependency, requiring large, labeled datasets for training. In fault scenarios such as micro-fractures or thermal-induced blade damage, these images are difficult to obtain. Future research aims to develop robust vision algorithms capable of operating under variable lighting and contamination conditions, possible through adaptive illumination and image enhancement techniques. Use of energy-efficiency edge computing platforms and lightweight AI models will be critical for real-time processing without excessive hardware costs. Another promising direction is multi-modal sensor fusion, combining vision data with thermal, acoustic emission, and vibration signals to improve diagnostic reliability. Advances in hyperspectral imaging and 3D vision systems can provide richer data for material characterization and blade geometry analysis. Digital twin integration will allow virtual simulations of blade wear and fracture progression using real-time vision feedback, enabling predictive maintenance at scale. Lastly, research into self-calibrating and self-cleaning optical systems enhances durability while reducing the maintenance overhead, paving the way for fully autonomous, Industry 4.0-enabled cutting operations. Vision-based approaches provide direct and interpretable wear information; however, their effectiveness is strongly constrained by environmental robustness. Many reported methods assume stable lighting and clean optical paths, conditions that are difficult to maintain in production sawing environments. In addition, the reliance on large, labeled image datasets limits scalability and cross-machine transferability.

## 5. Eddy-Current (EC) Sensors

**Figure 7:**
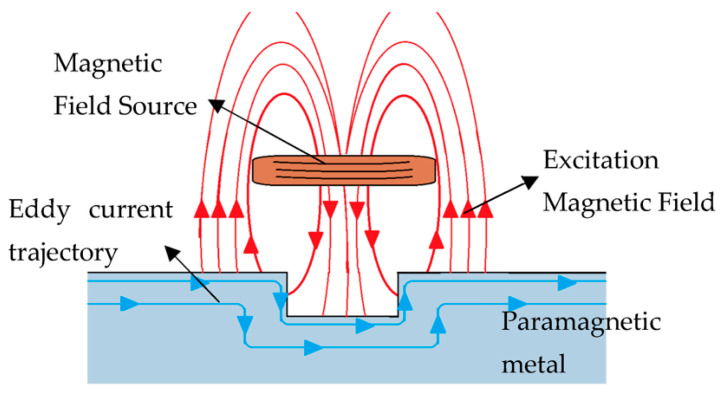
A representation of the currents induced by a magnetic field within an imperfect metallic material [75].

Eddy-current (EC) sensors constitute a robust class of non-contact electromagnetic sensing technologies extensively employed for defect detection, displacement measurement, and structural health monitoring in electrically conductive materials [73]. Their operation is governed by Faraday’s law of electromagnetic induction, whereby an alternating current passing through an excitation coil generates a time-varying magnetic field that interacts with a nearby conductive surface. This interaction induces localized circulating currents referred to as eddy currents within the material, whose magnitude and spatial distribution depend on the material’s electrical conductivity, magnetic permeability, geometry, and the presence of surface or subsurface discontinuities [73,74]. The induced eddy currents, in turn, generate secondary magnetic fields that oppose the original excitation field (Figure 7), resulting in measurable changes in the coil’s electrical impedance (resistance and inductance). Eddy current sensing measures changes in coil impedance caused by induced circulating currents within the conductive blade material, which depend on electrical conductivity and magnetic permeability. These impedance variations enable detection of subsurface fatigue, thermal softening, and microstructural evolution that cannot be observed optically. By analyzing these impedance variations, EC sensors can infer critical parameters such as lift-off distance, surface condition, material degradation, and hidden defects, including micro-cracks and fatigue damage. In cutting applications, components are subjected to high rotational speeds, cyclic mechanical loading, thermal fluctuations, and abrasive contact, all of which accelerate wear, crack initiation, and fatigue-driven failure mechanisms. Conventional inspection approaches, such as visual, vibration, or acoustic-based monitoring, are often limited in their ability to detect early-stage or subsurface defects, particularly during in-process operation. In contrast, EC sensing offers a real-time, non-destructive, and contactless monitoring solution capable of identifying incipient damage beneath the surface without halting production. These attributes make EC sensors suited for in situ health monitoring and predictive maintenance frameworks, aligning closely with data-driven, condition-based monitoring paradigms central to Industry 4.0 and smart manufacturing environments.

**Figure 8:**
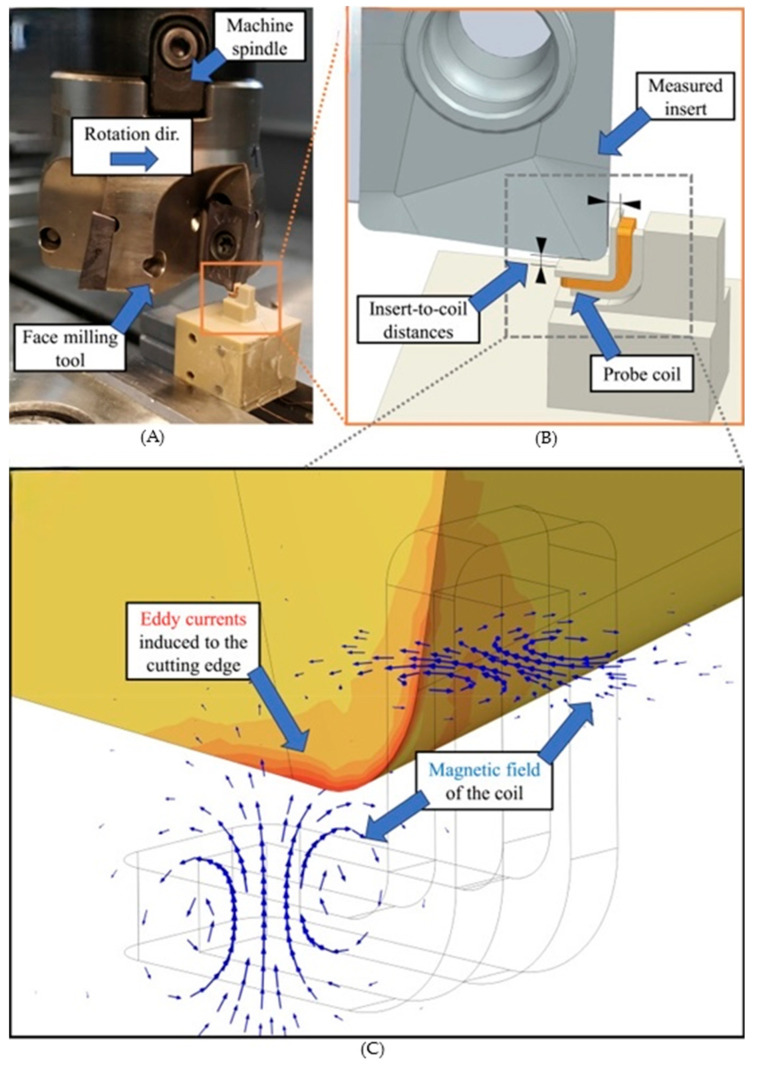
Overview of the experimental setup and eddy-current sensing methodology used to characterize cutting tool dynamics during face milling. (**A**) Photograph of the face-milling configuration showing the machine spindle, cutting tool, and direction of rotation during machining. (**B**) Schematic of the eddy-current probe arrangement relative to the cutting insert, highlighting the insert-to-coil distances and the measured insert geometry. (**C**) Numerical illustration of the electromagnetic sensing principle, showing the magnetic field generated by the probe coil and the resulting eddy currents induced in the cutting edge [21].

Recent frameworks have significantly advanced the role of EC sensors from classical non-destructive testing toward inline and high-resolution condition monitoring of conductive components across cutting contexts (Figure 8). Building on foundational EC principles, Kinnunen and Viitala introduced dedicated EC sensor designs for direct tool condition monitoring, demonstrating that changes in sensor coil impedance reliably correlate with progressive wear on milling and turning inserts, thereby establishing a real-time, non-contact approach to tool wear assessment in machining environments typical of bandsaws and other cutting systems [21]. Extending this work, the authors demonstrated the applicability of EC sensors to turning inserts, where impedance signatures enabled differentiation between wear, chipping, and fracture states under industrial cutting conditions [76]. At the sensor design level, Zhang et al. developed optimized planar EC probe geometries that significantly enhanced sensitivity to small fatigue cracks in aluminum alloys, a contribution particularly relevant for detecting early-stage crack initiation in high-speed cutting environments [77]. EC sensing has also been successfully applied to wear measurement under harsh operating environments. Park et al. used EC displacement sensors to monitor disc cutter wear under varying chamber pressures and debris conditions, confirming the robustness of EC measurements in environments analogous to industrial sawing operations [78]. Machado’s review of EC probe design gives insight into recent advances in differential coils, flexible substrates, and miniaturized probe architectures, emphasizing their role in improving spatial resolution, lift-off tolerance, and noise suppression for defect detection [74]. Analytical methods for EC signal interpretation have also evolved. For example, efficient predictive models for EC probe output in the presence of fatigue cracks provide a theoretical framework for understanding signal variations due to defect geometry and material properties [79]. In parallel, flexible EC array systems have been developed for surface and subsurface crack detection in welded steel structures, showcasing how multi-channel and bendable coil configurations enhance crack detection sensitivity and adaptability to complex surface geometries; qualities beneficial for blade health monitoring [80]. The integration of machine learning into EC inspection, as demonstrated by Sahu et al., illustrates how EC signal features can be automatically classified with high accuracy using random forests, paving the way for automated, data-driven flaw detection in industrial inspection workflows [81]. Sensor array innovations like the planar flexible differential fractal EC sensor further extend detection capability to micro-cracks as small as sub-millimeter scale, improving sensitivity for surface defect characterization [82]. Additional studies extend EC methodologies to detect subsurface flaws in additively manufactured materials, highlighting how probe geometry and excitation parameters can be tuned for specific defect types, which is informative for designing blade or cutting-edge monitoring systems [83]. EC parameter optimization for defect detection in AM parts likewise stresses the importance of tuning sensor frequency and geometry to balance penetration depth and signal fidelity. Jointly, the literature illustrates how EC sensors’ advanced coil designs, array integration, theoretical signal models, and intelligent data analysis are transitioning from traditional NDT toward real-time, in-process condition monitoring.

Eddy-current (EC) sensing offers non-contact, real-time monitoring of tool wear, blade integrity, and early-stage defects in conductive materials, making it a key technology for predictive maintenance in smart manufacturing. Despite its strengths as a non-contact, real-time monitoring technique for detecting defects and wear in conductive components, EC sensing has inherent limitations that constrain its performance in complex manufacturing environments. Eddy-current sensing offers unique access to subsurface damage, but its applicability remains restricted to conductive materials and carefully controlled lift-off conditions. Most studies focus on probe design and signal sensitivity, while fewer address long-term stability and robustness in dynamic cutting environments. Comparative validation against optical or force-based methods is also limited. A primary challenge is lift-off sensitivity, where variations in the gap between the probe and the material surface caused by surface unevenness, vibration, or coatings significantly reduce signal amplitude and can mask or distort defect signatures, thereby compromising detection reliability [84,85]. Surface condition effects, including roughness and cleanliness, further perturb EC responses by altering current flow patterns and lowering signal-to-noise ratios, making it difficult to distinguish true defects from benign variations [86]. Another fundamental constraint is limited penetration depth due to the skin effect, where eddy currents decay exponentially with depth; this restricts EC’s ability to reliably detect deep subsurface flaws unless excitation frequency, coil geometry, or probe design are carefully optimized [74,83]. Material properties such as electrical conductivity and magnetic permeability also have a significant influence, meaning EC techniques are most effective within a narrow range of conductive materials and less so for heterogeneous or layered structures. Additionally, complex geometries, edges, and curvature can introduce geometric distortion into EC signals, requiring specialized probe designs and calibration. Interpretation of EC measurements remains non-trivial; overlapping effects from lift-off, material variability, and noise necessitate advanced signal processing and often skilled operators to accurately differentiate defect characteristics. When applied in dynamic environments such as high-speed cutting operations, these limitations are exacerbated, particularly where consistent probe positioning and stable contact are difficult to maintain. Together, these challenges highlight the importance of multi-modal sensing strategies and ongoing research into adaptive probe designs, intelligent compensation algorithms, and hybrid inspection systems to overcome EC’s intrinsic constraints and expand its applicability in real-world manufacturing monitoring [87].

## 6. Force Sensors

**Figure 9:**
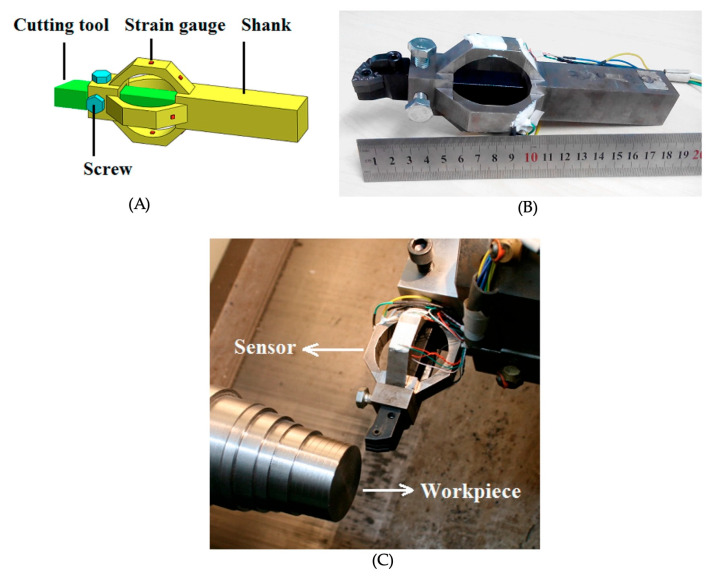
Strain-gauge-based cutting force sensing system for machining applications: (**A**) schematic illustration of a tool-integrated strain-gauge force sensor mounted on the tool shank; (**B**) photograph of the fabricated force sensor assembly with integrated sensing elements; and (**C**) experimental setup showing the force sensor installed on the machine tool during cutting, with the sensor measuring interaction forces between the cutting tool and the workpiece [92].

Cutting force sensors directly quantify the mechanical interaction forces at the tool–workpiece interface, providing a fundamental modality for assessing tool condition, process stability, and cutting efficiency. Unlike vibration, acoustic emission, or electromagnetic techniques, which infer cutting conditions indirectly, force sensors deliver direct, physically meaningful measurements and are often used to validate other monitoring approaches. Common systems include piezoelectric dynamometers, strain gauge-based transducers, load cells, and integrated force-measuring tool holders. Piezoelectric sensors provide high sensitivity and wide frequency bandwidth, while strain gauge sensors offer robustness and long-term stability (Figure 9) [88,89]. Multi-axis dynamometers can simultaneously measure tangential cutting force, feed force, and radial or thrust force [90,91]. Changes in these forces, both average values and transient spikes, can indicate tool wear, edge rounding, chipping, material inhomogeneity, or variations in friction at the tool–workpiece interface. In multi-tooth processes such as circular sawing or bandsawing, force signals often show periodic patterns corresponding to cyclic tooth engagement, providing insight into tooth load distribution, blade sharpness, cutting efficiency, and the onset of unstable cutting conditions.

Recent advances in cutting force measurement sensing have expanded both the hardware capabilities and analytical frameworks used for direct monitoring of cutting process conditions and tool wear. Song et al. developed a smart tool holder with integrated semiconductor strain gauges and neural network-based temperature compensation for accurate main cutting force acquisition during turning, demonstrating high resolution and dynamic stability close to the cutting zone, which improves the fidelity of force-based condition indicators in practice [93]. Plogmeyer et al. reported on tool-integrated thin-film sensor systems that measure both forces and temperatures through embedded structures, enabling compact force sensing with minimal modification to the cutting tool and improved multi-directional force capture during machining [94]. In milling operations, the integration of cutting force and temperature data from instrumented milling heads has been shown to enhance tool wear state estimation across break-in, steady-state, and accelerated wear regimes, illustrating the value of high-quality force signals for multi-condition diagnostics [95]. A study by Ahmad and Shah introduced a newly designed 3-axis force sensor tailored for lathe machines, offering improved spatial resolution of tangential, feed, and thrust components that underpin force-based monitoring frameworks in turning applications [96]. In dynamic milling contexts, PVDF thin-film sensors integrated into instrumented working tables have been used to capture force profiles that correlate with tool runout and tooth passing frequency, demonstrating alternative compact sensing substrates with cross-validated dynamometer fidelity [97]. By integrating a custom-built piezoelectric pressure sensor into the bandsaw’s architecture, Rakurty and Rangasamy shifted the perspective of force measurement from simple magnitude tracking to a detailed diagnostic of the tool–workpiece interface [98]. This specialized setup allowed for the identification of subtle force fluctuations and vibration signatures, providing a direct window into how lubrication rather than just cooling stabilizes the cutting process during the high-impact entry and exit phases of interrupted sawing. Beyond direct measurement, Chehrehzad et al. proposed a sensorless monitoring method that estimates cutting forces and torque using spindle current and torque data recorded on an industrial edge device, achieving real-time estimation with low error and pointing toward reduced dependency on costly dynamometers [99]. Rotational turning force measurement systems designed to analyze chip removal and feed versus passive force under varying kinematics have provided insight into complex kinematics where conventional force measurement is challenged, emphasizing that unique process dynamics require tailored force sensing solutions [100]. Predictive modeling studies have also addressed cutting force variability under different cutter–workpiece engagement stages, highlighting advanced modeling techniques that improve force predictability and interpretation across frequency components related to tooth passing and wear evolution [101]. Together, these recent contributions illustrate a trend toward integrated, sensor-based tool systems, alternative force sensing materials, and data-driven and sensor-less estimation models that enhance the robustness, practicality, and resolution of cutting force measurement sensing for tool condition monitoring across diverse machining operations.

Cutting force measurement sensors provide direct, real-time monitoring of mechanical loads at the tool–workpiece interface, offering valuable insight into tool condition, process stability, and cutting efficiency. Despite their ability to measure tangential cutting force, feed force, and radial or thrust force, these sensors face inherent limitations that constrain their performance in complex machining environments. High-precision dynamometers are often costly and require careful mechanical integration, which can alter system stiffness or interfere with normal operation. Thermal drift, calibration sensitivity, and structural compliance can also contaminate the force signal, reducing measurement fidelity. In multi-tooth processes such as circular sawing or bandsawing, distributed cutting edges and rotating blades complicate direct force measurement, while transient variations in cutting engagement may mask localized tool damage or material inhomogeneity. Additionally, the physical interpretation of force signals, such as associating increased average forces with wear progression, edge rounding, or friction changes can be confounded by noise, vibration, or irregular workpiece geometry. To address these challenges, future work could explore miniaturized and lower-cost sensor designs, improved thermal and calibration compensation, and advanced signal-processing algorithms. Integrating cutting force measurements with complementary sensing modalities through multi-sensor fusion can enhance robustness, enable predictive maintenance, and improve the reliability of tool condition monitoring in real-world production environments.

## 7. Hybrid/Multi-Modal Sensors

**Figure 10:**
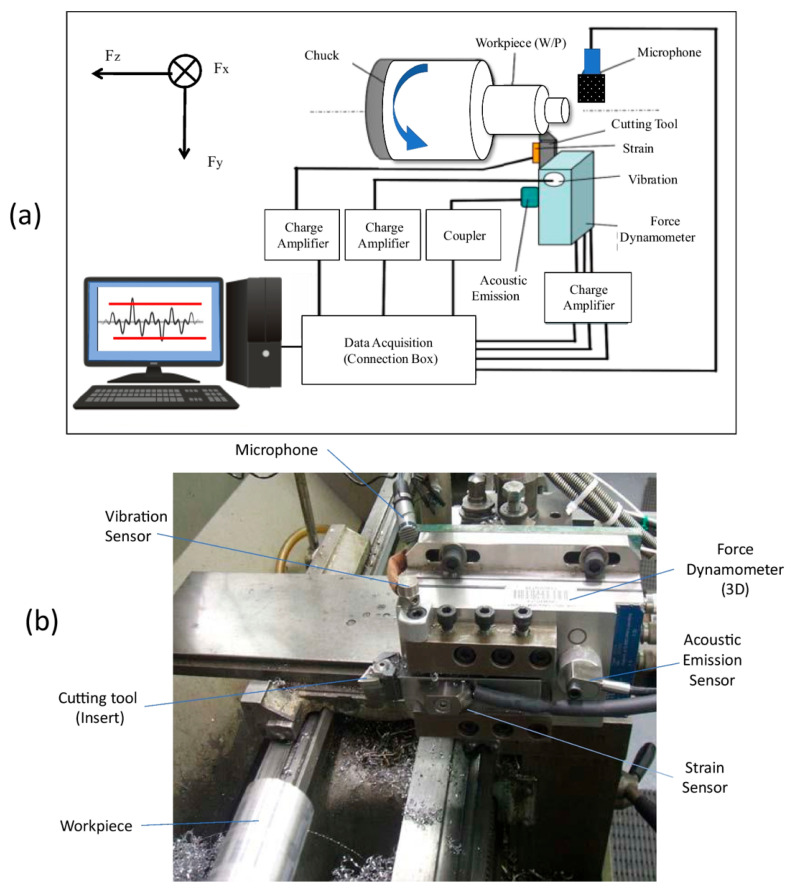
Experimental setup for multi-sensor machining process monitoring: (**a**) Schematic representation of the data acquisition system, illustrating the integration of force, vibration, acoustic emission, strain, and sound pressure sensors; (**b**) Photographic view of the experimental turning configuration showing the physical placement of the sensors relative to the cutting tool and workpiece [102].

Hybrid or multimodal sensing refers to the coordinated use of multiple distinct sensor modalities to observe complementary aspects of a physical process, thereby enabling a more comprehensive and robust representation of system behavior than any single sensor. In cutting and machining applications, this typically involves the simultaneous acquisition and analysis of signals such as vibration, acoustic emission, cutting force, eddy current, and vision-based measurements, which are then combined through data-level, feature-level, or decision-level fusion strategies (Figure 10). By leveraging the strengths of different sensing principles while mitigating their individual limitations, hybrid sensing frameworks are particularly well suited for monitoring complex, non-stationary cutting environments where tool–workpiece interactions, machine dynamics, and environmental variability can confound single-modality diagnostics. As a result, multimodal sensing has become an increasingly important enabler for reliable tool condition monitoring, predictive maintenance, and intelligent control within Industry 4.0-oriented manufacturing systems.

Recent advances in intelligent machining and cutting process monitoring increasingly emphasize hybrid and multimodal sensing approaches, where multiple sensor modalities such as vibration, acoustic emission, cutting force, motor current, and vision are jointly analyzed to improve robustness, accuracy, and reliability of tool condition monitoring under non-stationary operating conditions. A central motivation for this trend is that single-sensor measurements often suffer from ambiguity, noise sensitivity, or limited observability of complex cutting phenomena, whereas sensor fusion frameworks exploit complementary signal characteristics to mitigate individual sensor limitations. For example, recent milling studies demonstrate that fusing vibration, cutting force, and spindle motor current signals using deep learning architectures such as ResNet–LSTM significantly improves tool wear prediction accuracy compared to single-modality inputs [103]. Similarly, multi-sensory fusion strategies combining accelerometers, electrical current sensing, strain gauges, and machine vision have been validated in turning operations, where unsupervised clustering and machine-learning-based classifiers achieved more stable wear state recognition under varying cutting conditions [104]. Layered or hierarchical fusion architectures integrating acoustic emission, vibration, and force measurements have further shown strong predictive capability for flank wear estimation in CNC machining, supporting the use of ensemble learning and multi-level feature fusion to handle heterogeneous sensor data streams [105]. Vision-augmented hybrid approaches have also gained traction, where image-based features extracted from tool or surface inspection are fused with vibration and force signals to enhance remaining useful life (RUL) prediction and wear progression modeling, particularly in thin-walled or high-precision machining scenarios [106]. In parallel, multimodal sensing frameworks combining vibration, sound, power consumption, and acoustic emission signals have been applied to detect chatter initiation and classify wear states in cutting and sawing processes, highlighting the ability of hybrid approaches to capture both dynamic and energetic signatures of tool–workpiece interactions [59]. Additional studies integrate optical sensing with conventional signal-based monitoring to overcome the indirect nature of purely vibration or force-based inference, enabling more interpretable diagnostics and improved real-time decision-making [107]. Beyond data-level fusion, hybrid sensing has also been embedded within expert systems and digital monitoring platforms for precision turning, where fused sensor inputs inform adaptive control strategies and automated fault diagnosis [108]. From a systems perspective, recent work further demonstrates that hybrid sensor fusion combined with edge–cloud or IoT architectures enables scalable, real-time monitoring across diverse machining environments, reinforcing its relevance to Industry 4.0-enabled cutting systems [109]. Reviews and comparative studies of multimodal fusion featuring vibration, acoustic emission, and force signals outperform single-sensor approaches in wear prediction accuracy, robustness to noise, and generalization across tools and materials [110,111]. Collectively, these studies indicate that hybrid sensing and sensor fusion are emerging as a key enabler for reliable, data-driven condition monitoring in cutting applications, particularly as manufacturing systems transition toward autonomous, AI-assisted operation.

While multi-sensor fusion has shown clear performance advantages over single-modality monitoring in cutting applications, its practical deployment still faces several unresolved technical and operational challenges. A main issue is the increased system complexity caused by integrating diverse sensors with different bandwidths, sampling rates, noise profiles, and spatial reference frames. This complicates synchronization, calibration, and long-term stability in industrial settings. These issues are amplified in rotating or high-speed cutting systems, where sensor drift, timing misalignment, and electromagnetic interference can significantly bias fused data and reduce diagnostic accuracy. Additionally, multi-sensor fusion systems usually entail higher hardware, integration, and maintenance costs, raising barriers for small and medium manufacturers and limiting scalability beyond lab tests. From a data analytics perspective, fusion methods often rely on high-dimensional feature spaces and machine learning models that require a lot of data, increasing susceptibility to overfitting and reducing robustness when labeled training data are sparse or operating conditions differ from those used during model development. Many studies show performance improvements under controlled conditions, but their ability to generalize across tools, materials, and machines remains under-validated. Possible solutions include developing standardized sensing architectures and adaptive synchronization. calibration methods, physics-informed feature selection, and hierarchical or feature-level fusion schemes that reduce data dimensionality and computational burden. Emerging edge-AI and embedded processing platforms further offer pathways to limit latency and bandwidth requirements while enabling real-time inference. Looking ahead, future research must move beyond performance benchmarking toward self-adaptive fusion frameworks that dynamically reweight sensor contributions based on process context, and toward tighter integration of hybrid sensing data with digital twins for predictive control and decision-making. Addressing these limitations will be essential for transitioning multi-sensor fusion from promising research prototypes to robust, cost-effective solutions suitable for widespread adoption in Industry 4.0-enabled cutting systems. Table 1 compiles the sensing modalities addressed in this review and contrasts their operational principles, application domains, advantages, limitations, measurement challenges, and relative costs within modern cutting-process monitoring frameworks.

Beyond sensing performance, practical deployment of sensor technologies in cutting environments is strongly influenced by measurement difficulty and total system cost, including sensor hardware, integration, data acquisition, and signal processing. Measurement difficulty refers to how easily a sensing modality can produce repeatable and reproducible measurements during cutting operations, where “low” indicates measurements are both repeatable and reproducible with minimal calibration or environmental control, “moderate” indicates repeatable measurements can be achieved but reproducibility may require controlled conditions or additional calibration, and “high” indicates measurements that are difficult to repeat and not reliably reproducible across different machines, cutting conditions, or sensor setups. Vibration sensors, particularly MEMS accelerometers, offer a low-cost (<$2000) and relatively simple solution with minimal installation requirements, making them attractive for widespread industrial adoption. Acoustic emission systems, while offering superior sensitivity to early-stage damage, involve medium–high total cost ($8000–20,000) associated with high-frequency transducers, signal conditioning, and data processing infrastructure, as well as greater complexity in sensor placement and noise mitigation. Vision-based sensing systems vary widely in cost, ranging from low-cost (<$2000) camera solutions to high-cost (>$20,000) laser or hyperspectral systems, and often require controlled lighting, calibration, and substantial computational resources. Eddy-current sensors fall within a medium cost range ($2000–$8000) but require careful probe design, calibration, and control of lift-off distance, particularly in dynamic cutting environments. Force sensors, such as piezoelectric or strain-gauge-based dynamometers, provide direct measurement of cutting forces and are highly relevant to tool condition monitoring and process optimization; however, they can be medium–high to high cost (~$8000 to >$20,000) and often require careful mounting, calibration, and protection from harsh cutting environments, which can complicate integration. Hybrid and multi-modal sensing systems generally incur the highest cost (>$20,000) and integration complexity due to the need for synchronization, data fusion, and advanced analytics, but they offer superior robustness and diagnostic reliability. These trade-offs highlight that sensor selection must balance performance benefits against economic feasibility and deployment complexity for specific cutting applications.

## 8. Conclusions

This mini-review examined recent advances in sensing technologies applied to condition monitoring, predictive maintenance, and process optimization in cutting operations, with particular emphasis on vibration, acoustic emission, optical and vision-based, eddy-current, and hybrid multi-modal sensing approaches. Collectively, the reviewed studies demonstrate a clear transition from isolated, single-sensor monitoring toward integrated, data-driven frameworks capable of capturing the complex and non-stationary dynamics inherent in cutting processes. While vibration and acoustic emission sensors remain effective for detecting dynamic responses and early fault signatures, their susceptibility to noise and operating-condition variability motivates complementary sensing strategies. Optical and vision-based systems provide direct geometric and surface information, and eddy-current sensors enable non-contact detection of surface and subsurface defects, each addressing distinct diagnostic gaps but also introducing modality-specific constraints. Force sensors, including piezoelectric and strain-gauge dynamometers, provide direct measurement of cutting forces, offering highly relevant insights into tool wear, chatter, and process efficiency. While their installation and calibration can be more demanding compared to vibration or acoustic sensors, the direct force measurements they provide complement other modalities and enhance predictive capabilities. Increasingly, hybrid and multi-modal sensor fusion approaches are being adopted to leverage complementary information across sensing domains, improving robustness, reducing diagnostic ambiguity, and enabling more reliable inference under realistic manufacturing conditions. Despite these advances, challenges related to system complexity, data availability, computational demand, and interoperability persist and currently limit large-scale industrial adoption. Future research should prioritize scalable multi-modal fusion architectures, physics-informed and explainable AI models, and edge-enabled analytics that support real-time operation and generalization across machines and materials. The integration of fused sensing data within digital twin frameworks further presents a promising pathway toward adaptive control and autonomous decision-making. Overall, continued progress in sensor integration, data analytics, and system standardization is expected to play a central role in enabling intelligent, self-optimizing cutting systems aligned with Industry 4.0 manufacturing paradigms.

## Figures and Tables

**Table 1:** Sensor Modalities, Capabilities, and Limitations for Data-Driven Monitoring of Cutting Processes.

Sensing Modality	Sensing Principle	Measured Phenomena	Typical Applications	Key Advantages	Limitations	Industry 4.0 Relevance	Measurement Difficulty	Relative Cost
**Vibration sensors**	Measure oscillatory motion and convert mechanical vibrations into electrical signals using piezoelectric, capacitive, or MEMS elements	Acceleration, vibration amplitude, frequency response, resonance behavior	Tool wear detection, imbalance and misalignment identification, chatter detection, blade fatigue analysis	Non-intrusive, mature technology, high sensitivity to mechanical faults, well-suited for continuous monitoring	Signal noise and complexity, sensitivity to sensor placement, environmental effects, data-intensive ML models	Predictive maintenance, IoT-enabled monitoring, AI-based diagnostics, digital twin updating	Low-moderate	Low
**Acoustic emission (AE) sensors**	Detect high-frequency elastic stress waves generated by micro-crack initiation, plastic deformation, and frictional events	Ultrasonic transient signals, hit rate, signal energy, RMS amplitude	Early-stage tool wear detection, micro-crack identification, chatter onset monitoring, subsurface damage detection	Extremely sensitive to incipient damage, early fault detection, effective for continuous cutting	High data rates, complex signal interpretation, durability challenges, limited standardization	High-resolution condition monitoring, AI-enhanced fault classification, sensor fusion for autonomous systems	High (signal processing intensive)	Medium-high
**Optical/vision based sensors**	Use cameras, lasers, or structured light to capture images or 3D profiles of tools and workpieces	Tool geometry, edge wear, surface defects, dimensional deviations	Direct tool wear inspection, blade alignment monitoring, surface quality assessment, automated quality control	Non-contact, direct visual measurement, high spatial resolution, intuitive diagnostics	Sensitive to lighting, dust, coolant, motion blur; high computational demand; integration complexity	Automated inspection, AI-driven quality control, closed-loop process optimization, digital twin visualization	Moderate-high (lighting, computation)	Medium-high
**Eddy-current (EC) sensors**	Electromagnetic induction generates eddy currents in conductive materials; impedance changes indicate defects or wear	Surface and subsurface defects, crack initiation, displacement, material degradation	Subsurface crack detection, blade integrity monitoring, insert wear measurement	Non-contact, real-time operation, effective for conductive materials, detects hidden damage	Lift-off sensitivity, limited penetration depth, material dependency, complex calibration	Inline health monitoring, NDT integration into smart manufacturing, predictive maintenance frameworks	Moderate (lift-off sensitivity)	Medium
**Force Sensors**	Measure cutting forces and moments by converting mechanical loads into electrical signals using piezoelectric, strain-gauge, or capacitive transducers	Cutting force components (Fx, Fy, Fz), torque, force variation, load transients, specific cutting energy	Tool wear and breakage detection, chatter identification, process stability monitoring, adaptive feed and speed control	Direct physical insight into cutting mechanics, strong correlation with tool wear, suitable for closed-loop control, mature modeling	Intrusive integration, thermal drift, sensitivity to machine compliance, limited high-frequency response	Closed-loop machining control, adaptive manufacturing, physics-informed digital twins, predictive maintenance	Moderate	Medium–high
**Hybrid/Multi-modal sensors**	Integration of multiple sensing modalities (e.g., vibration, acoustic emission, force, current, vision) using data-, feature-, or decision-level fusion strategies	Combined dynamic, acoustic, visual, electromagnetic, and energetic signatures of the cutting process	Robust tool condition monitoring, wear progression tracking, chatter detection, and fault diagnosis under non-stationary cutting conditions	Enhanced diagnostic robustness, complementary information capture, reduced ambiguity compared to single-sensor approaches	Increased system complexity and cost, synchronization and calibration challenges, high data volume and computational demand	AI-driven predictive maintenance, autonomous machining through sensor fusion, digital-twin-enabled monitoring, adaptive Industry 4.0 manufacturing systems	High (integration and analytics)	High

## Data Availability

Not applicable.
